# Can’t find the antidote: A root cause analysis

**DOI:** 10.3389/fphar.2022.895841

**Published:** 2022-09-06

**Authors:** Laila Carolina Abu Esba, Ghada Mardawi, Mohammad Al Deeb

**Affiliations:** ^1^ King Abdulaziz Medical City, Ministry of National Guard, Health Affairs, Riyadh, Saudi Arabia; ^2^ King Saud Bin Abdulaziz University for Health Sciences, Riyadh, Saudi Arabia; ^3^ King Abdullah International Medical Research Centre, Riyadh, Saudi Arabia; ^4^ Pharmaceutical Care Services, King Abdulaziz Medical, Riyadh, Saudi Arabia; ^5^ Department of Emergency Medicine, Medical Toxicology division, King Abdulaziz Medical City, Riyadh, Saudi Arabia; ^6^ College of medicine, King Saud Bin Abdulaziz University for Health Sciences, King Abdullah International Medical Research Centre, Riyadh, Saudi Arabia

**Keywords:** antidotes, stocking, poisoning, methanol, fomepizol

## Abstract

**Background:** In a series of cases that came to be recognized as a national methanol outbreak, an incident of delay in allocation and treatment with the antidote fomepizole is described with aim of sharing a learning experience.

**Method:** A team of 16 members was formed to conduct a Root Cause Analysis (RCA), which included multiple individual interviews with the stakeholders and inspection visits to the area.

**Results:** Root causes: The restocking process was unclear and inconsistent and specifically lacked a restocking policy for antidotes, inappropriate labeling and area design, and a sound-alike between fomepizole and omeprazole. Contributing factors included: unsuitable restocking practice and lack of training in using the pharmaceutical electronic inventory system. Corrective actions were recommended and implemented.

**Conclusion:** Management of antidotes in large healthcare systems requires a team effort to ensure appropriate and timely availability in emergency poisoning cases. This RCA identified important areas for improvement that could be insightful to other institutions in preventing similar vulnerabilities and is unique in describing the details of system improvements that can have a large impact on patient safety.

## Background

Methanol toxicity is a serious poisoning that has high morbidity and mortality, which typically occurs in outbreaks in the community through ingestion of homemade or illegally produced alcoholic drinks. Despite that, the world health organization’s global report on health outcomes related to alcohol describes that methanol toxicity only accounted for less than 1% of all alcohol-attributable deaths (WHO, 2014). A study in the United States reported that the inpatient prevalence of methanol toxicity was 6.4 cases per 1,000,000 admissions. Nevertheless, these outbreaks are tragic events as methanol poisoning if not rapidly treated, causes retinal injury and eventually permanent blindness, as well as ischemic or hemorrhagic injury to the basal ganglia, renal failure, respiratory failure, and death (Kaewput et al., 2021).

In September 2020, we identified our first case of methanol toxicity which turned into a series of cases that came to be recognized as a national methanol outbreak, as other cases were reported at other hospitals. All these cases were linked to one source of poorly adulterated alcoholic beverages, distributed illegally. Familiarity with methanol toxicity is not common in our region. A retrospective review of methanol toxicity cases received over the past decade using data from our pharmacy showed that only 15 vials of fomepizole were dispensed over 10 years, with a zero consumption over the past 3 years ahead of this outbreak, compared to 95 vials utilized since the outbreak.

A literature search of methanol toxicity cases in Saudi Arabia (SA) revealed only four cases (Algahtani et al., 2018; Althwanay et al., 2020; Owolabi et al., 2020). However, Galvez-Ruiz et al. described outcomes of optic disk cupping following methanol poisoning in a series of 50 cases presenting at two large hospitals in Riyadh between 2008 and 2014, possibly indicating a higher incidence of methanol toxicity than perceived (Galvez-Ruiz et al., 2015). A recent study that used the United States National Inpatient Sample database reported 6.4 cases per 1,000,000 inpatient prevalence of methanol toxicity (Kaewput et al., 2021).

Insufficient stocking and availability of antidotes have been reported as a worldwide problem (Dart et al., 1996; Al-Sohaim et al., 2012; Gasco et al., 2013; Thanacoody et al., 2013). The Institute of Safe Medication Practice (ISMP-Canada) in a safety bulletin recognized the inaccessibility to antidotes and availability of information resources to guide timely use as main vulnerabilities in dealing with toxicity cases (Ismp-canada, 2018).

This paper aims to share a learning experience as we describe the incidence of delay in allocation and treatment with the antidote fomepizole in the first cases of methanol toxicity presented at our facility. We also describe the root cause analysis (RCA) of the incidence and the measures taken to ensure patient safety and prevent a recurrence.

No patient or case details are described in this paper, the aim is to share barriers to proper medication management of antidotes to promote patient safety.

## Event summary

In 2020, around the beginning of the autumn season and just as COVID-19 lockdown and restrictions on gatherings were eased in SA, the first case in a series of cases of methanol toxicity presented to the emergency room (ER) during the weekend. With classic signs and symptoms of methanol toxicity including metabolic acidosis and changes in the level of consciousness. The diagnosis was confirmed with methanol blood levels. At this point, dialysis and intravenous (IV) fomepizole were ordered.

The order for fomepizole was entered in the computerized physician order entry (CPOE) as a STAT order, which was verified by the ER satellite pharmacy within 2 mins; however, no stocks of fomepizole were available in the ER satellite pharmacy. The pharmacy technician proceeded to check the pharmacy’s electronic inventory system, which indicated that stocks were available in the pharmacy warehouse. The technician called and paged the on-call pharmacist in charge of the pharmacy main store but no one answered.

The pharmacy technician also called the intensive care unit (ICU) satellite pharmacy in another attempt to locate stocks of fomepizole, as satellite pharmacy stocks are not linked to the electronic inventory system, and thus, stocks need to be checked manually. Over the phone, the pharmacist misheard fomepizole IV as omeprazole IV, which is not a formulary item at the hospital, and therefore, the pharmacist provided a negative response, replying that no stocks are available. The technician also contacted the IV room pharmacy but they also had no stock.

Five hours from the initial order the on-call pharmacist in charge of the pharmacy main store answered, but replied that no stocks are available in the pharmacy warehouse.

The patient was then transferred to the ICU where a new order for fomepizole was made. The ICU pharmacy technician was also unable to locate the stock of fomepizole, but a senior pharmacy technician was aware of its presence. The first dose of fomepizole was finally administered to the patient 6 hours after the initial order.

**FIGURE 1:**
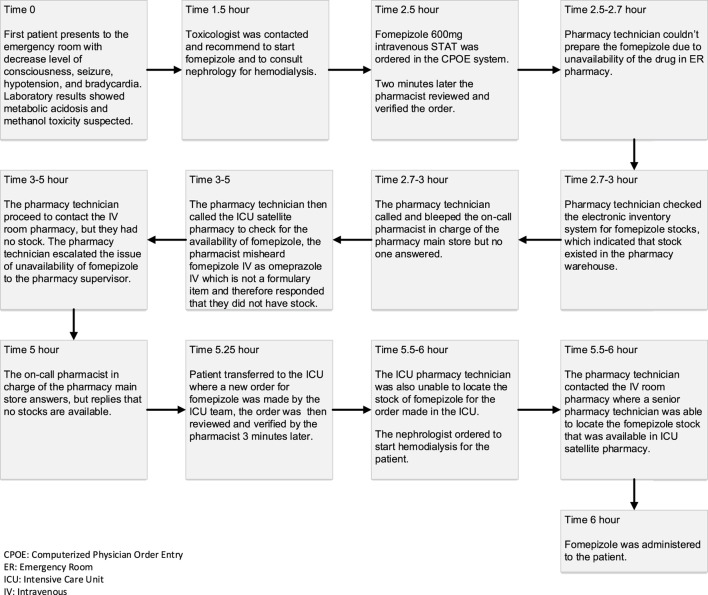
Timeline and sequence of the event.

During that time, the pharmacy director and director of pharmaceutical planning who were off-duty were informed by hospital staff about the unavailability of fomepizole and began efforts in borrowing stocks from other hospitals. The director of pharmaceutical planning requested from one of his off-duty staff to physically check the pharmacy warehouse for stocks of fomepizole, which were available and delivered to the ICU-satellite pharmacy within 7 hours from the initial fomepizole order for the index case. Figure 1 outlines the pharmaceutical distribution process at our institution.

**FIGURE 2:**
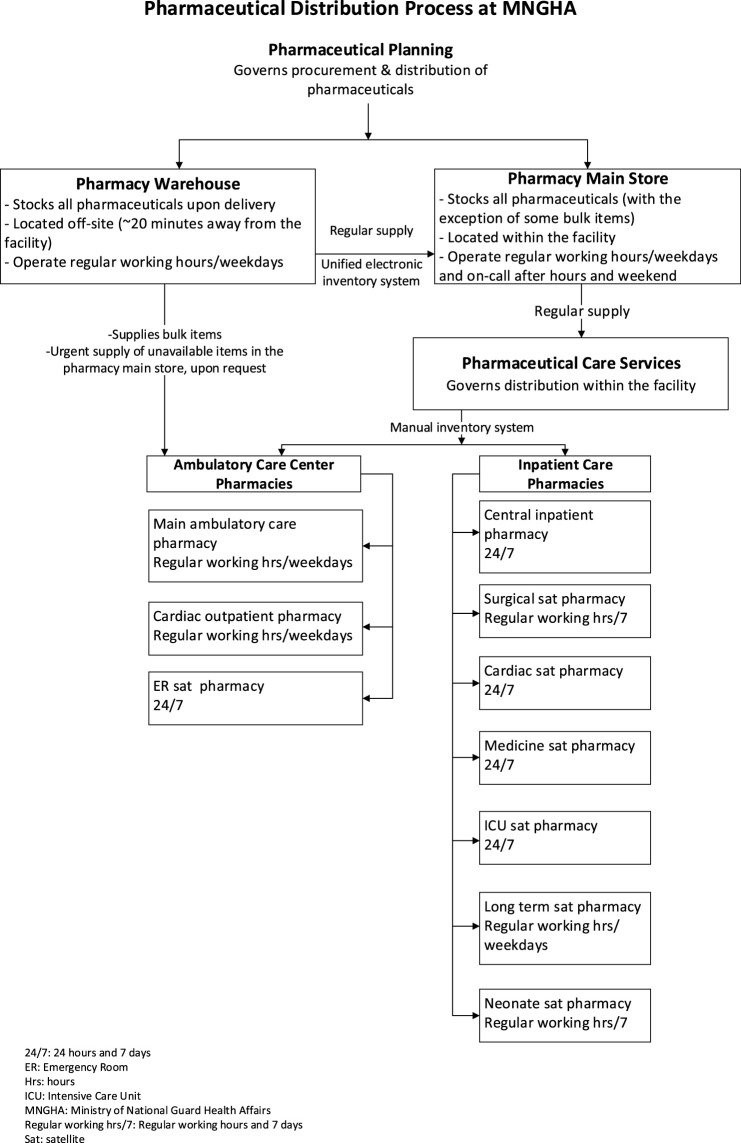
Pharmaceutical Distribution Process at MNGHA.

The toxicologists overseeing the patient had also inquired the pharmacy about the availability of ethanol (an alternative antidote for methanol toxicity), which was unfortunately not listed as a formulary item and was only stocked in the narcotic pharmacy in small quantities for other uses, and pharmacy staff were not aware of its availability. Figure 2 outlines the timeline and sequence of the event.

### Method

Setting: King Abdulaziz Medical City (KAMC) is a 1500-bed tertiary care hospital in Riyadh, Saudi Arabia, and is under the umbrella of the large integrated health system of the Ministry of National Guard Health Affairs, which includes six medical cities. The hospital has emergency services, specialized toxicology physicians, a drug and poison information center (DPIC) and its laboratory service is well equipped to perform all necessary toxicology testing.

The medication safety program is a well-established division of the quality and patient safety department at KAMC, and is responsible for monitoring and analyzing all reported medication adverse events, regularly conducts RCA and identifies areas for improvement, and recommends actions to prevent patient harm. All recommendations are discussed in a collective corporate meeting including all regional hospitals, and agreed-upon recommendations are implemented in all hospitals.

### The root cause analysis process

The delay in allocation and treatment of patients presenting with methanol toxicity was discussed in our pharmacy and therapeutics committee and a memo was sent to our quality and patient safety department to officially conduct an RCA and respond to our committee with results for improvement and actions to be taken.

A team of 16 members was formed to conduct the RCA and included: medication safety officers (team leader), quality improvement specialists, ER consultant, a toxicologist, a nursing medication safety representative, the DPIC pharmacist, director of the pharmaceutical planning department, and the director of warehousing and distribution. In addition to the main RCA meeting, multiple individual interviews with the stakeholders and inspection visits to the area were conducted.

The institutional review board (IRB) at King Abdullah International Medical Research Center (KAIMRC) (Protocol NRC21R/520/12) approved this study. No consent form was required by the Ministry of National Guard Health Affairs ethics committee.

## Results of the RCA

The event was deemed preventable by the task force evaluating the incidence. The family was disclosed about the event.

The RCA elucidated the fundamental error in this event to be the failure to locate the available stocks of fomepizole within the hospital by pharmacy staff, resulting in a 6-hours delay in the administration of the antidote. On further analysis, this error of medication administration delay was attributed to the following three root causes:

### Root-causes identified

1) Distribution of Supplies Factors: Before the event occurred the ER satellite pharmacy requested stocks of fomepizole from the pharmacy warehouse; however, as the request was submitted on the last business day (i.e. before the weekend), the ER pharmacy was not re-supplied, which resulted in a zero stock over the weekend. The pharmacy main store did not stock all medications and fomepizole as a low-consumed drug was not stored. In addition, the pharmacy technician was unaware of the process of responding to zero stock issues and escalating it to the supervisors. The restocking process was unclear and inconsistent and specifically lacked a restocking policy for antidotes including fomepizole injection, with no clear guidance on communication with senior staff in an emergency situation.
2) Work Environment Factors: The allocated space for the ICU satellite pharmacy was small, with limited shelves and space, as a result, fomepizole as a drug rarely dispensed, was stored on a lower and not so visible shelf. It was also stored following no specific alphabetic or therapeutic class order (e.g., antidote shelf), which made it impossible for the junior staff to find, and only the senior pharmacy technician knew where it was stocked. Therefore, the ICU satellite pharmacy was unable to supply the ER satellite pharmacy with the available stocks. Hence, the root cause of this deviation was inappropriate area design.
3) Communication factors: The sound-alike between fomepizole and omeprazole led the ICU pharmacist to inform the ER pharmacy technician that fomepizole was not available in stock; this was a verbal miscommunication over the phone. The delay in response from the on-call pharmacist in the pharmacy main store and the misinformation about the availability of stocks in the pharmacy warehouse led the rest of the efforts to focus on borrowing from other hospitals, while stocks were available in the pharmacy warehouse.

### Contributing factors

1) Distribution of Supplies Factors: The system of restocking implemented by pharmaceutical planning was based on the average monthly usage (AMU), which in cases of medications with unclear trends in consumption (i.e. no average monthly use) required a justification from the pharmacy technician to be approved for restocking. This led the first request sent from the ER satellite pharmacy for fomepizole stocks to be rejected, while the second request was approved just before the weekend but not executed. The contributing factor to this deviation was guidelines do not enable one to carry out the task promptly.
2) Education & Training: The lack of competency of pharmacy staff in using the pharmaceutical electronic inventory system resulted in incorrect information about stock availability.

### Root‐cause analysis: Recommendations & improvements

After multiple meetings with the stakeholders and parties involved the RCA team agreed on a list of recommendations, and the team is continuously working on these recommendations and has been able to implement changes to improve patient safety. Table 1 summarizes these recommendations and improvements achieved. Table 2 lists the antidotes added to the formulary after the RCA.

**TABLE 1:** Summary of the RCA Recommendations and Improvements Achieved.

**Root‐cause analysis Recommendations**	**Improvements Achieved**
**Workflow**	
Develop a new workflow between the pharmaceutical care department, pharmaceutical planning, and the facility’s ER satellite pharmacy on requesting medications unavailable in the pharmacy main store.	The workflow of ordering and re-stocking antidotes between the pharmacy main store and the ER & ICU satellite pharmacy was developed.
Move all antidotes from the warehouse to the pharmacy main store immediately upon delivery (excluding antidotes with non-toxicological indications and regular consumption e.g. Calcium chloride).	All antidotes are now stocked in the pharmacy main store upon delivery (i.e. on-site).
**Storage & Workspace**	
Dedicate a special area within the ER and ICU satellite pharmacies for stocking antidotes (antidote shelves), including designated shelves in the refrigerator for refrigerated antidotes.	We now have a specific area within the ER and ICU satellite pharmacies for storing antidotes. All antidotes are stored in a clear area on the antidote shelf in alphabetical order with an information sheet on the minimum quantity available for each antidot to be checked and verified by staff on each shift.
Expand the ICU satellite pharmacy to accommodate the huge service covered by the satellite.	Planned and under consideration.
**Pharmaceutical Planning**	
Develop a task force to estimate the minimum quantity required for all antidotes, and provide that list to the planning department for procurement.	The team identified important antidotes that were not listed on the formulary. Overall, ten antidotes were evaluated and approved by the hospital’s pharmacy and therapeutics committee for addition. See table 2 for the list of antidotes added. The team developed the hospital's antidote list with the minimum quantity that should be stocked at the facility, utilizing published literature, real historical consumption data, the expertise of toxicologists dealing with toxicity cases, together with i
**Education**	
Provide staff with easy access to antidote information (e.g. indication, dosing, and administration).	At the time of this publication, the team had developed 20 antidote data sheets, which include information about the antidote’s indication, adult and pediatric dosing, method of preparation, monitoring parameters, and administration. These data sheets were also posted on the hospital’s intranet to ensure rapid access to information, and are also accessible in hard copies on the antidote shelves.
**Technical**	
To expedite and support the new electronic integrated pharmaceutical inventory module which will also be integrated with the CPOE system.	Under development.

CPOE: computerized physician order entry; ER: Emergency Room; ICU: Intensive Care Unit

**TABLE 2:** List of Antidotes on the Formulary before and after the RCA.

**Formulary Antidotes Before**	**Formulary Antidotes After**	**Indication**
Acetylcysteine injection	Acetylcysteine injection	Acetaminophen toxicity
NA	Andexanet alfa injection	Reversal of anticoagulation for patients treated with a direct factor Xa inhibitor (apixaban or rivaroxaban)
Atropine injection	Atropine injection	Organophosphate pesticide or nerve agent poisoning, carbamate toxicity
Calcium Chloride 10% prefilled syringe	Calcium Chloride 10% prefilled syringe	Fluoride, calcium channel blocking agent toxicity
NA	Calcium gluconate gel 2.5%	Hydrofluoric acid dermal burns
Calcium gluconate injection 10%	Calcium gluconate injection 10%	Fluoride, calcium channel blocking agent toxicity
Cyproheptadine tablet	Cyproheptadine tablet	Serotonin toxicity
Dantrolene sodium injection	Dantrolene sodium injection	Malignant hyperthermia
Deferoxamine mesylate injection	Deferoxamine mesylate injection	Iron poisoning
Digoxin immune fab injection	Digoxin immune fab injection	Cardiac glycosides toxicity or cardiac steroid toxicity
Dimercaprol injection (BAL in oil) injection	Dimercaprol injection (BAL in oil) injection	Heavy metal toxicity (arsenic, lead, mercury)
NA	Edetate calcium disodium/EDTA ampule	Lead poisoning
NA	Ethanol dehydrated alcohol injection	Methanol or ethylene glycol poisoning
Flumazenil injection	Flumazenil injection	Benzodiazepine toxicity
Fomepizole injection	Fomepizole injection	Methanol or ethylene glycol poisoning
Glucagon prefilled syringe	Glucagon prefilled syringe	β-blocker, calcium channel blocker toxicity
Hydroxocobalamin injection	Hydroxocobalamin injection	Cyanide poisoning
NA	Idarucizumab injection	Reversal of anticoagulant effects of dabigatran
L-carnitine vials	L-carnitine vials	Valproic acid toxicity
Leucovorin calcium vial	Leucovorin calcium vial	Methotrexate or methanol toxicity
Methylene blue injection	Methylene blue injection	Methemoglobinemia, ifosfamide induced encephalopathy
Naloxone injection	Naloxone injection	Opioid toxicity
Octreotide vial	Octreotide vial	Sulfonylurea-induced hypoglycemia
NA	Pentetate calcium trisodium injection (Calcium DTPA)	Internal contamination with plutonium, americium, or curium to increase the rates of elimination
Phentolamine mesylate injection	Phentolamine mesylate injection	Extravasation
Physostigmine injection	Physostigmine injection	Anticholinergic syndrome
Phytonadione injection	Phytonadione injection	Reversal of coumarin-induced coagulopathy
Phytonadione tablet	Phytonadione tablet	Reversal of coumarin-induced coagulopathy
Polyvalanet snake antivenom injection	Polyvalanet snake antivenom injection	Snake envenomation
Polyvalent scorpion antivenom injection	Polyvalent scorpion antivenom injection	Scorpion envenomation
Potassium iodide tablet	Potassium iodide tablet	Thyroid radioiodine protection
Pralidoxime chloride injection	Pralidoxime chloride injection	Organophosphorus poisoning
Protamine sulfate injection	Protamine sulfate injection	Reversal of coagulopathy induced by unfractionated or low-molecular-weight heparin
NA	Prussian blue capsule	Thallium or radiocesium toxicity
Pyridoxine tablet	Pyridoxine tablet	Isoniazid or hydrazine toxicity
Regular human insulin vial	Regular human insulin vial	Beta-blocker toxicity, calcium-channel blocker toxicity
Smoflipid 20% lipid injectable emulsion	Smoflipid 20% lipid injectable emulsion	Local anesthetic systemic toxicity
Sodium bicarbonate injection 8.4% injection	Sodium bicarbonate injection 8.4% injection	Tricyclic antidepressant toxicity, urine alkalization for salicylate toxicity, or cocaine toxicity
NA	Sodium Thiosulphate injection	Extravasation
NA	Succimer DMSA capsule	Heavy metal toxicity (arsenic, lead, mercury)
Sugammadex injection	Sugammadex injection	Reversal of neuromuscular blockade
Thiamine hydrochloride injection	Thiamine hydrochloride injection	Ethylene glycol toxicity, thiamine deficiency associated with chronic alcoholism
Unactivated prothrombin complex concentrates 4 factors II, VII, IX, X, with proteins S and C injection	Unactivated prothrombin complex concentrates 4 factors II, VII, IX, X, with proteins S and C injection	Reversal of anticoagulant bleeding
NA	uridine triacetate 10gram/packet oral granules	Fluorouracil or capecitabine overdose regardless of symptoms or early-onset toxicity

NA: Not available on the formulary

## Discussion

Planning pharmaceutical inventory can be extremely challenging in such a dynamic setting of disease trends, drug discovery, and new clinical research, adding to these, the rarity of some indications and their therapeutic utilization makes it more challenging.

The outbreak of COVID-19 in 2020 and the shutdown of major pharmaceutical plants around the world added to the challenge of a well-planned secured drug supply in many healthcare settings (Abu Esba et al., 2020).

Poisoning cases vary in epidemiology and often lack good evidence on incidence, in addition to geographical differences in types and frequencies of occurrences. This makes local assessment and decisions on antidote stocking crucial (Azab et al., 2016; Sandilands and Bateman, 2016).

A national survey of antidote stocking in the United Kingdom revealed that atropine, calcium gluconate, and flumazenil were the only antidotes available in adequate stocks in all hospitals. They also reported that only (24.3%) of the hospitals held all antidotes that were recommended to be available immediately, which only improved to (47.9%) after auditing and introducing a national guideline. They concluded that more efforts are required to ensure timely access to other antidotes (Bailey et al., 2016).

This RCA triggered a holistic review of our antidote preparedness and identified areas of weakness in stocking and access that were important to be reassessed and modified to be prepared for any further poisoning cases presenting. Similar efforts have been shared by the Nova Scotia antidote program which demonstrated that adequate stocking is achievable through ongoing surveillance and maintenance by a multidisciplinary team (Murphy et al., 2019).

Pharmacists working in poison centers should be able to provide information on the appropriate use of antidotes including method of preparation, dosing, and monitoring in addition to advice on procurement and stocking of antidotes suitable to the size and area of their served community.

Cultural aspects and difference also unique to our setting was the unavailability of ethanol, which in other countries is readily available. Therefore, antidote stocking and planning should be tailored to address these differences.

Whatever the reasons for inadequate stocking of antidotes, be it cost, availability, infrequent use, or lack of awareness, institutions should make all efforts to regularly review and audit their antidote stocks.

## Conclusion

Management of antidotes in large healthcare systems requires a team effort to ensure appropriate and timely availability in emergency poisoning cases. This RCA identified important areas for improvement that could be insightful to other institutions in preventing similar vulnerabilities. It provides details on operational level modifications that are needed to ensure safe access to antidotes when needed. The implemented measures require future analysis and assessment of their success in improving access to antidotes.
